# Supplementary material for: High-throughput screening identifies Aurora kinase B as a critical therapeutic target for Merkel cell carcinoma
Source: Nat Commun. 2025 Feb 12;16:1583. doi: 10.1038/s41467-025-56504-7 (PMC11822212; doi:10.1038/s41467-025-56504-7)
Supplement: Supplementary file 1 — Supplementary Information [file 41467_2025_56504_MOESM1_ESM.pdf]

High-throughput screening identifies Aurora kinase B as a critical therapeutic target for Merkel cell carcinoma

Supplementary Materials:

Supplementary Fig. S1. Area under the dose-response curve (AUC) histograms for primary small molecule screen.

Supplementary Fig. S2. MCC selective compounds.

Supplementary Fig. S3. VP-MCC and VN-MCC cell lines cluster separately in response to drug treatment.

Supplementary Fig. S4. VP-MCC and VN-MCC selective drug target subcategories relative to controls.

Supplementary Fig. S5. Follow-up validation screen correlates with primary high-throughput small molecule screen.

Supplementary Fig. S6. Aurora kinase inhibitors more potently reduce viability in VP-MCC than VN-MCC.

Supplementary Fig. S7. AURKB transcript is expressed in human MCC tumor samples.

Supplementary Fig. S8. AURKB signaling partners in the CPC are differentially regulated in VP-MCC relative to VN-MCC.

Supplementary Fig. S9. Biochemical profiling of aurora kinase inhibitors identified AZD2811 as a potent AURKB inhibitor.

Supplementary Fig. S10. AURKB inhibitor AZD2811 induces mitotic dysregulation in MCC.

Supplementary Fig. S11. AURKB inhibitor AZD2811 induces apoptosis in MCC.

Supplementary Fig. S12. AURKB inhibitor AZD2811 has no effect on MCPyV-LT expression in VP-MCC cell lines.

Supplementary Fig. S13. AZD2811NP tumor growth responses and pharmacokinetic profile.

Supplementary Fig. S14. AURKB inhibitor AZD2811NP reduces H3Ser10 phosphorylation and Ki-67 expression in MCC13 xenografts.

Supplementary Fig. S15. AURKB is a central hub connecting MCPyV oncogenes, the CPC, and stathmin-1.

Supplementary Dataset 1. Compounds demonstrating activity in all MCC, VP-MCC, or VN-MCC cell lines, but not in control cell lines.

Supplementary Dataset 2. List of pan-MCC, VP-MCC, and VN-MCC selective compounds relative to controls.

Supplementary Dataset 3. Results of arrayed RNAi druggable genome screen in MKL-2 and MCC26 cells.

Supplementary Dataset 4. MIPE library compound target subcategory annotation.

Supplementary Dataset 5. Summary of high-throughput small molecule and RNAi screens.

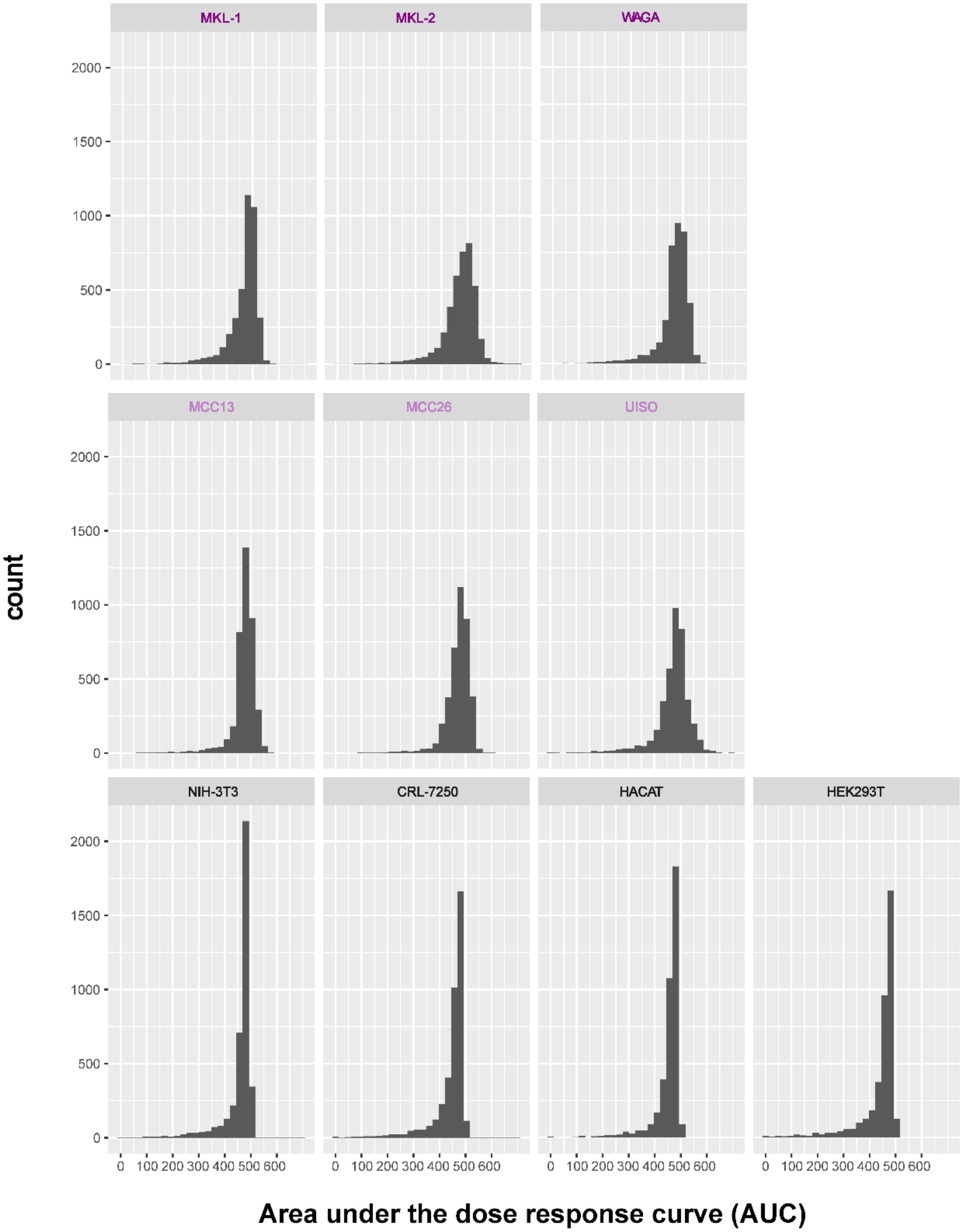

Supplementary Fig. S1. Area under the dose-response curve (AUC) histograms for primary small molecule screen. AUC histograms showing the distribution of drug responses for VP-MCC (top), VN-MCC (middle), and control cell lines (bottom). Median AUC for each cell line was MCC13: 482, MCC26: 480, UISO: 483, WAGA: 479, MKL-1: 483, MKL-2: 482, HACAT: 469, HEK293T: 466, NIH-3T3: 476, CRL-7250: 466. n = 1, indicating the number of experimental replicates for each cell line.

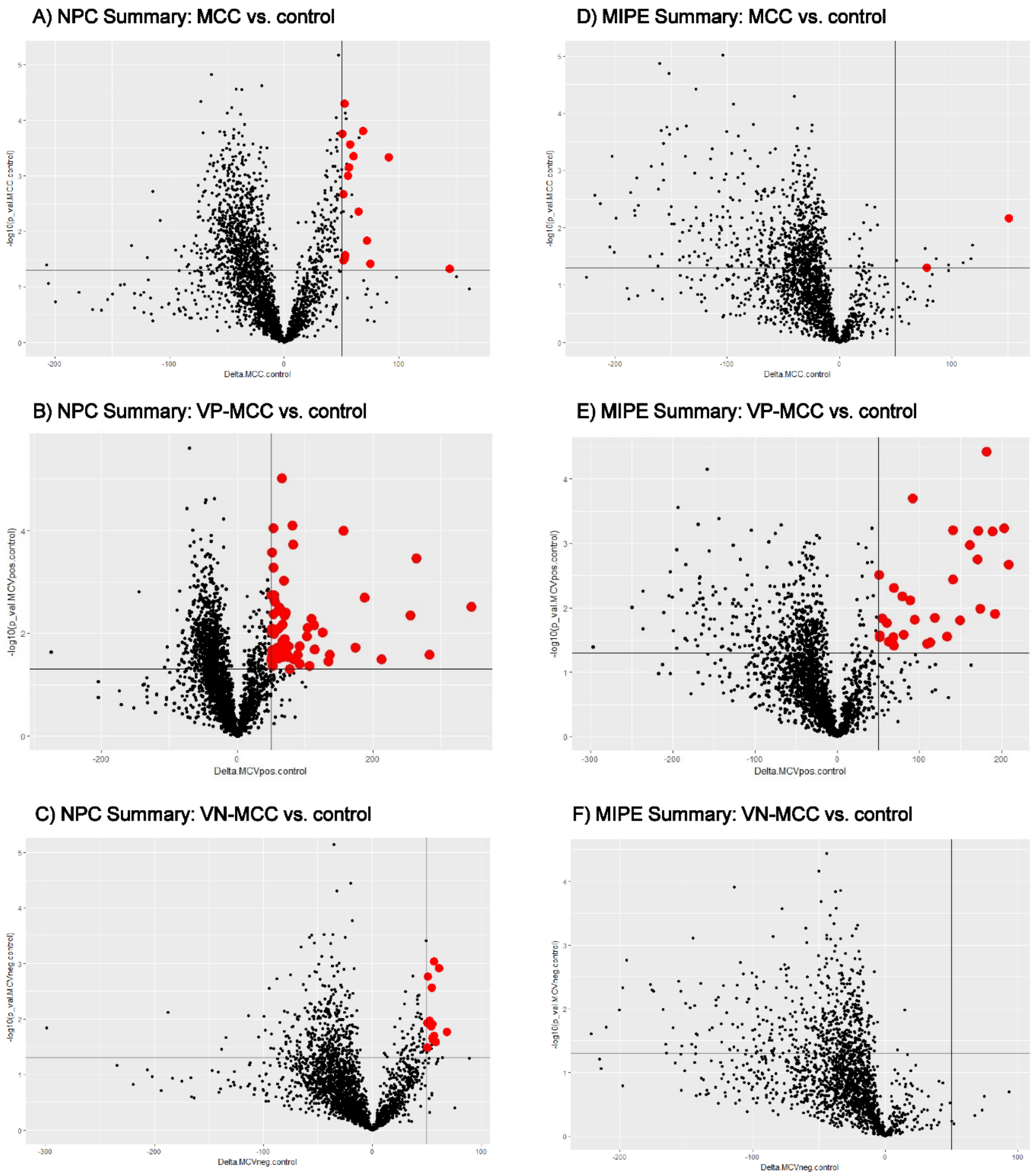

Supplementary Fig. S2. MCC selective compounds. Volcano plots illustrating MCC-selective compounds from the NPC or MIPE library. For each compound, the difference between average control cell line AUC (HACAT, HEK293T, CRL-7250, and NIH-3T3) and average MCC AUC (WAGA, MKL-1, MKL-2, MCC13, MCC26, and UISO) (A, D) or average VP-MCC AUC (WAGA, MKL-1, and MKL-2) (B, E) or average VN-MCC AUC (MCC13, MCC26, and UISO) (C, F) was calculated and plotted against the -$\log_{10}$ p-values for corresponding cell populations. Red points indicate an AUC difference > 50 (Control-MCC > 50), p-value < 0.05, and AUC for all MCC cell lines/VP-MCC/VN-MCC < 450. Compound names can be found in Supplementary Dataset 2. n = 1, indicating the number of experimental replicates.

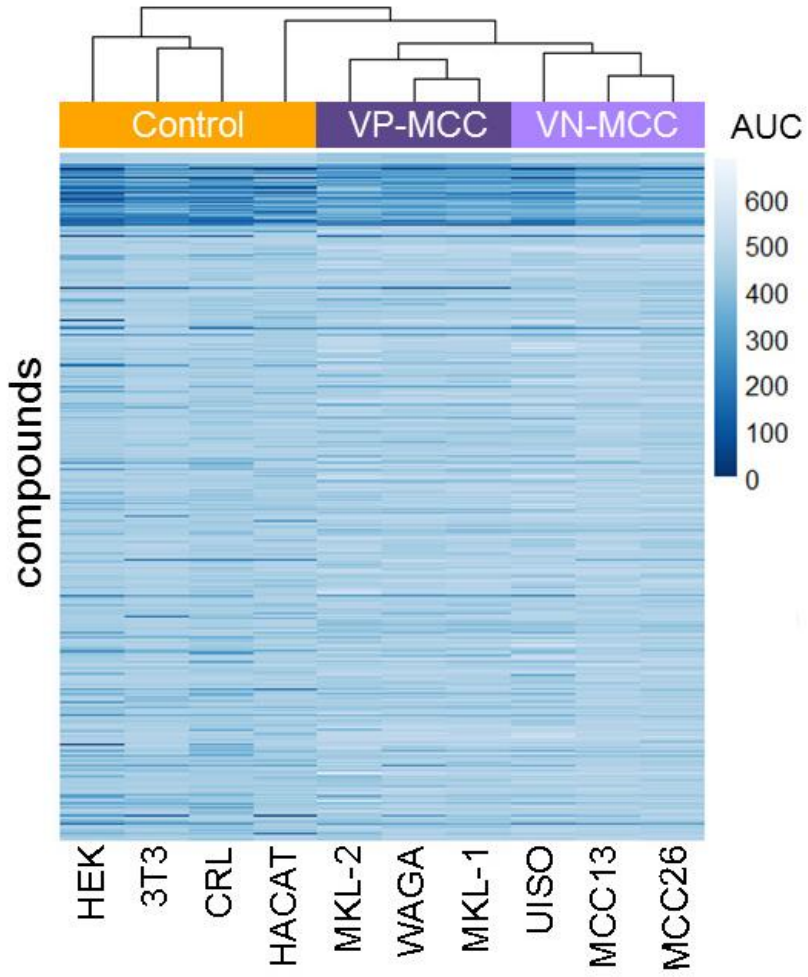

Supplementary Fig. S3. VP-MCC and VN-MCC cell lines cluster separately in response to drug treatment. Hierarchical clustering of 2546 compounds that reduced viability in at least one cell line (AUC < 450). Heatmap shows AUC (darker blue indicates more potent and efficacious). n = 1, indicating the number of experimental replicates for each cell line.

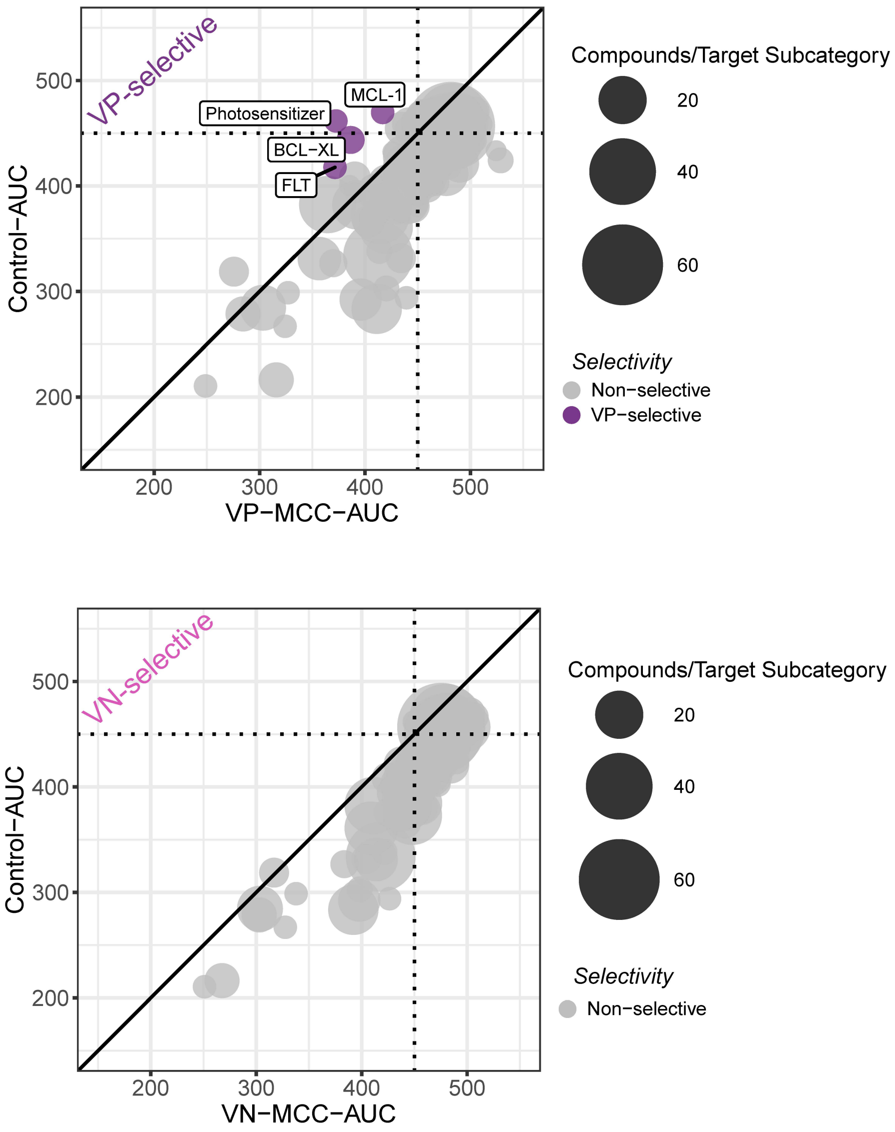

Supplementary Fig. S4. VP-MCC and VN-MCC selective drug target subcategories relative to controls. Bubble plots demonstrating average VP-MCC-AUC (A) or VN-MCC-AUC (B) vs. Control-AUC for each target subcategory (size of bubble is proportional to the number of agents in the target subcategory). A) Purple bubbles illustrate target subcategories that are selectively associated with VP-MCC viability (AUC difference > 40, $p < 0.05$, AUC < 450 in all VP-MCC cell lines screened). P-values: BCL-XL = 0.0014, FLT = 0.0139, MCL-1 = 0.0322, Photosensitizer = 0.00066. B) There were no VN-MCC selective target subcategories. n = 1, indicating the number of experimental replicates.

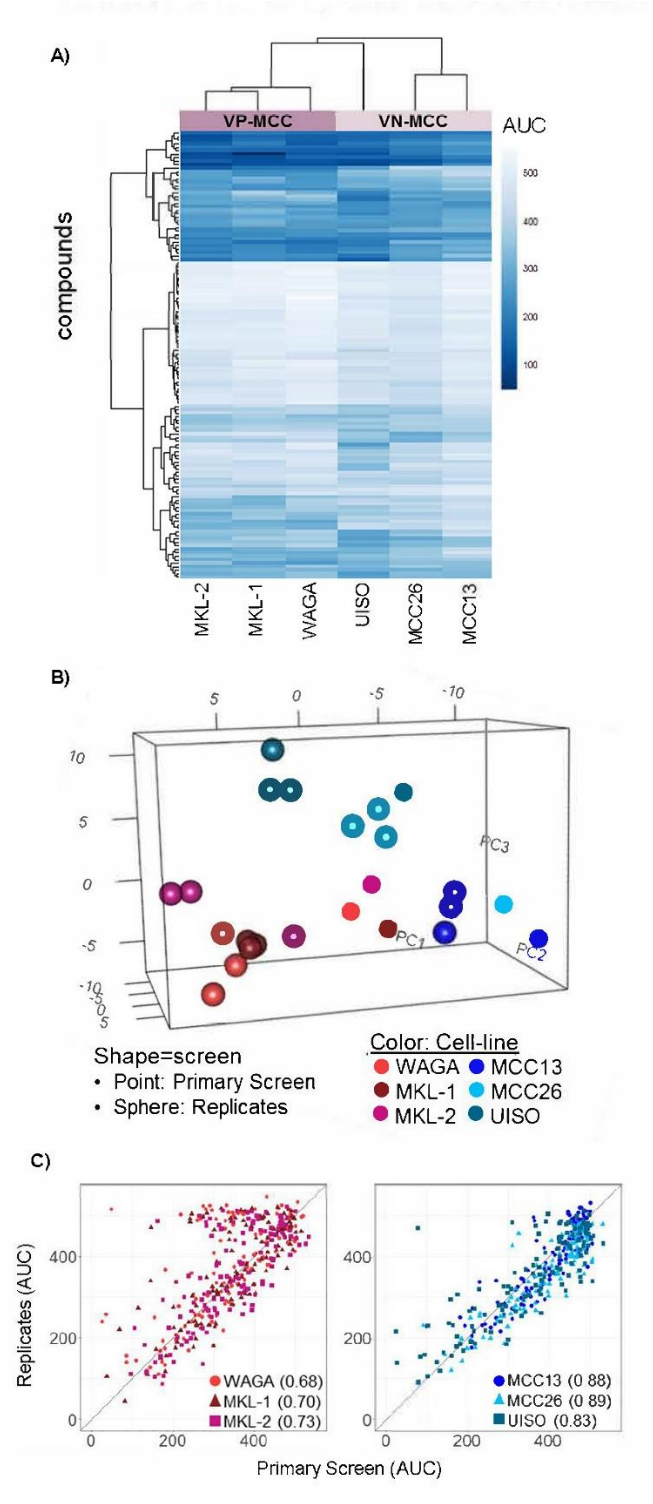

Supplementary Fig. S5. Follow-up validation screen correlates with primary high-throughput small molecule screen. A) Heatmap showing average AUC (blue-scale) for all compounds (n = 128, indicating the number of compounds) that were included in the follow-up screen (n = 3, indicating the number of experimental replicates for each cell line). B) PCA and C) Pearson correlation coefficients demonstrate precision across high-throughput (n = 1) and follow-up (n = 3) screens for all MCC cell lines. For B and C, n = number of experimental replicates for each cell line.

A)

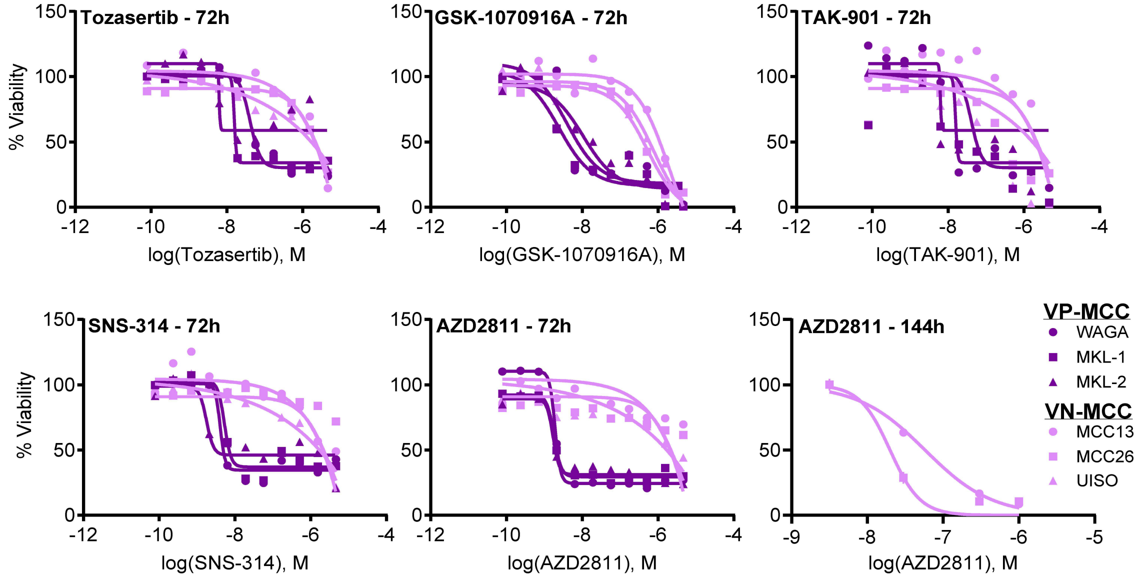

B)

AURK inhibitor IC50 (M)					
Cell line \ Compound	AZD2811	Tozasertib	GSK-1070916A	SNS-314	TAK-901
WAGA - 72h	*1.91e-9*	*4.04e-8*	*3.90e-9*	*4.09e-9*	*6.68e-9*
MKL-1 - 72h	*1.80e-9*	*1.57e-8*	*2.44e-9*	*5.29e-9*	*2.23e-8*
MKL-2 - 72h	*1.69e-9*	*6.24e-9*	*1.06e-8*	*1.77e-9*	*1.03e-8*
MCC13 - 72h	*1.11e-7*	*ND*	*1.98e-6*	*4.16e-6*	*2.40e-2*
MCC26 - 72h	*7.41e-7*	*3.62e-6*	*5.29e-7*	*2.01e-5*	*2.27e-7*
UISO - 72h	*ND*	*ND*	*7.711e-7*	*ND*	*1.110e-7*
MCC13 - 144h	*5.76e-8*				
MCC26 - 144h	*1.98e-8*				

C)

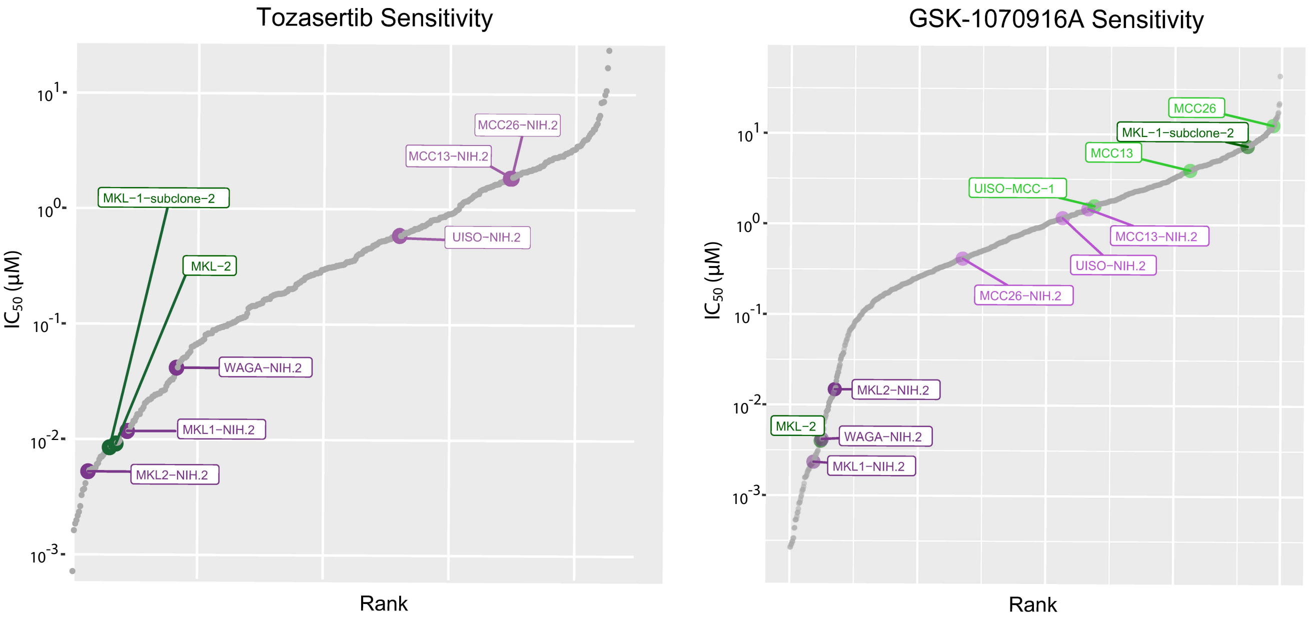

Supplementary Fig. S6. Aurora kinase inhibitors more potently reduce viability in VP-MCC than VN-MCC. A) Representative aurora kinase inhibitor dose-response curves for AZD2811, tozasertib, SNS-314, TAK-901, and GSK-1070916A. Cell viability was measured by CellTiter-Glo at 72 h and by manual cell counts at 144 h. Dose-response curves were fit in GraphPad Prism using either three or four-parameter logistic equations. Consistent across n = 3 replicates . B) IC_{50} values (molar) for each aurora kinase inhibitor in each MCC cell line. C) IC_{50} values (micromolar) from GDSC for tozasertib in 420 cell lines (left) and for GSK-1070916A from 950 cell lines (right). IC_{50} values from GDSC (Gray-scale and green) were combined with IC_{50} values determined in our study (purple) and then ranked. n = number of experimental replicates for each cell line.

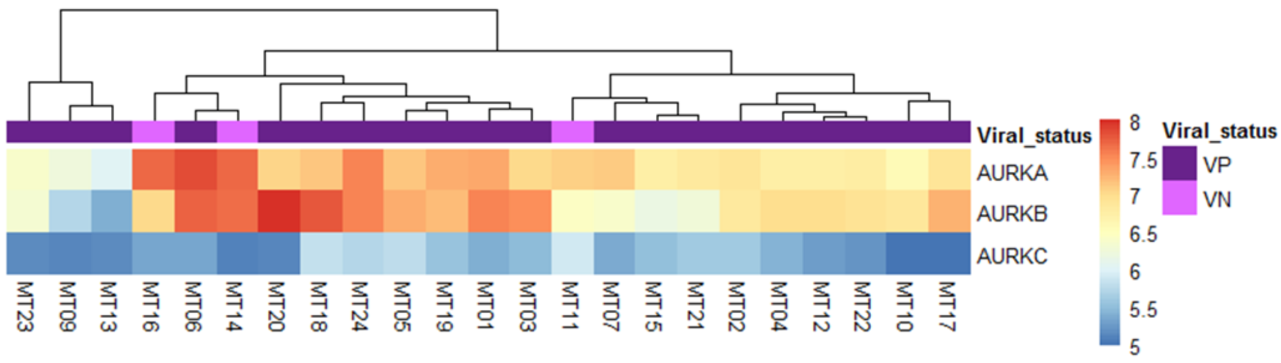

Supplementary Fig. S7. AURKB transcript is expressed in human MCC tumor samples. Transcript expression measured by microarray for AURKA, AURKB, or AURKC in 23 human MCC samples[19]. VP-MCC and VN-MCC samples are coded by dark or light purple, respectively.

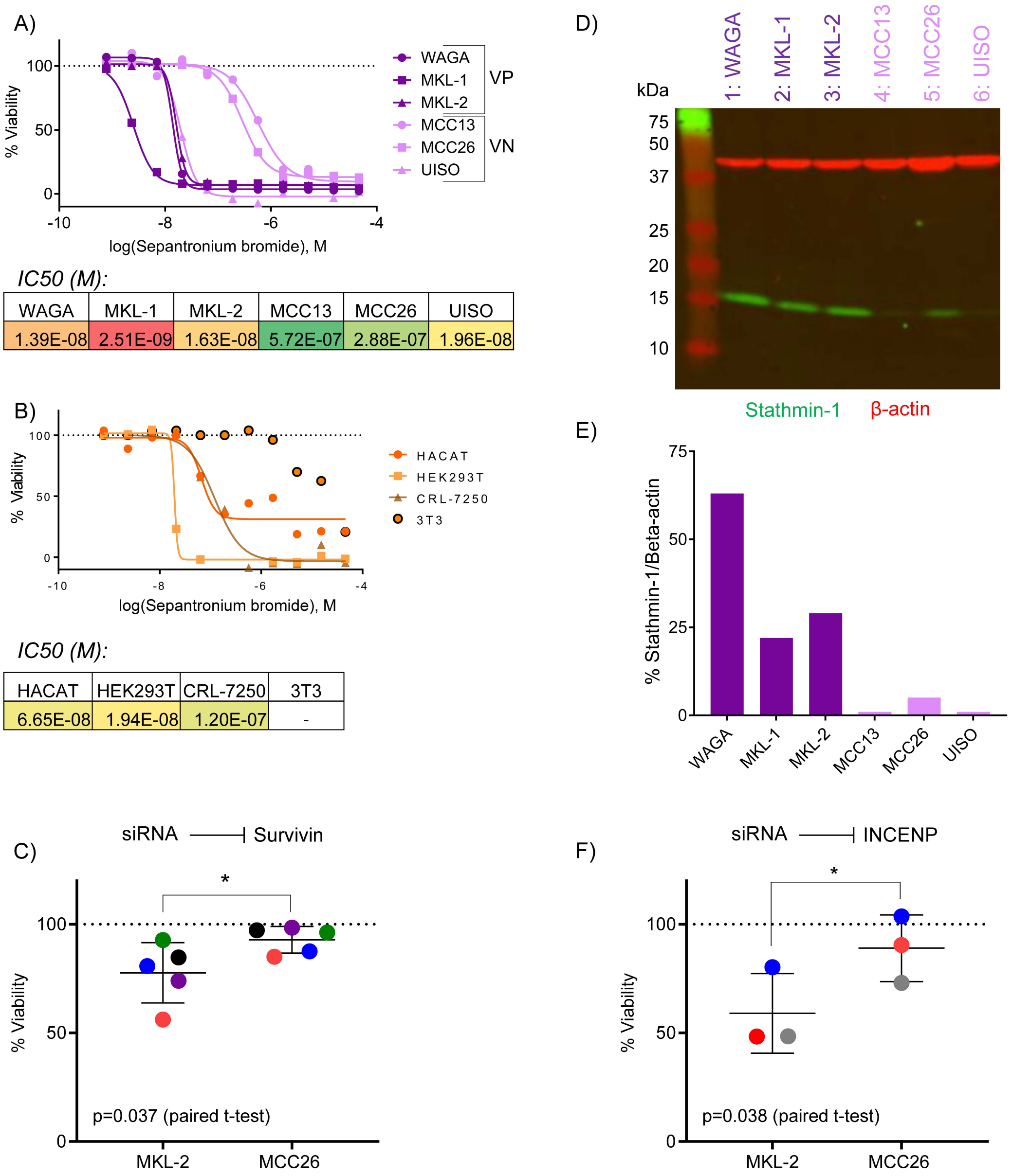

IC50 (M):

WAGA	MKL-1	MKL-2	MCC13	MCC26	UISO
1.39E-08	2.51E-09	1.63E-08	5.72E-07	2.88E-07	1.96E-08

IC50 (M):

HACAT	HEK293T	CRL-7250	3T3
6.65E-08	1.94E-08	1.20E-07	-

Supplementary Fig. S8. AURKB signaling partners in the CPC are differentially regulated in VP-MCC relative to VN-MCC. A/B) Dose-response curves for the survivin inhibitor sepantronium bromide in VP-MCC (A), VN-MCC (A), and control (B) cell lines (n = 1). Tables indicate the IC_{50} (molar) for sepantronium bromide calculated in GraphPad Prism using a four-parameter logistic equation. C) Knockdown of survivin (color indicates siRNA; n = 5) reduces viability in MKL-2 cells relative to MCC26 cells (p = 0.037, measured by a paired two-sample t-test). D) Representative Western Blot demonstrating higher stathmin-1 protein expression in VP-MCC cell lines (WAGA, MKL-1, and MKL-2) than VN-MCC cell lines (MCC13, MCC26, and UISO). E) Stathmin-1 protein expression (green) normalized to β-actin loading control (red) from representative Western Blot demonstrates higher stathmin-1 protein expression in VP-MCC than VN-MCC (consistent across n = 3 replicates). F) Knockdown of INCENP (color indicates siRNA; n = 3) reduces viability in MKL-2 cells relative to MCC26 cells (p = 0.038, measured by a paired two-sample t-test). For A, B, D, and E, n = number of experimental replicates. For C and F, n = number of siRNAs.

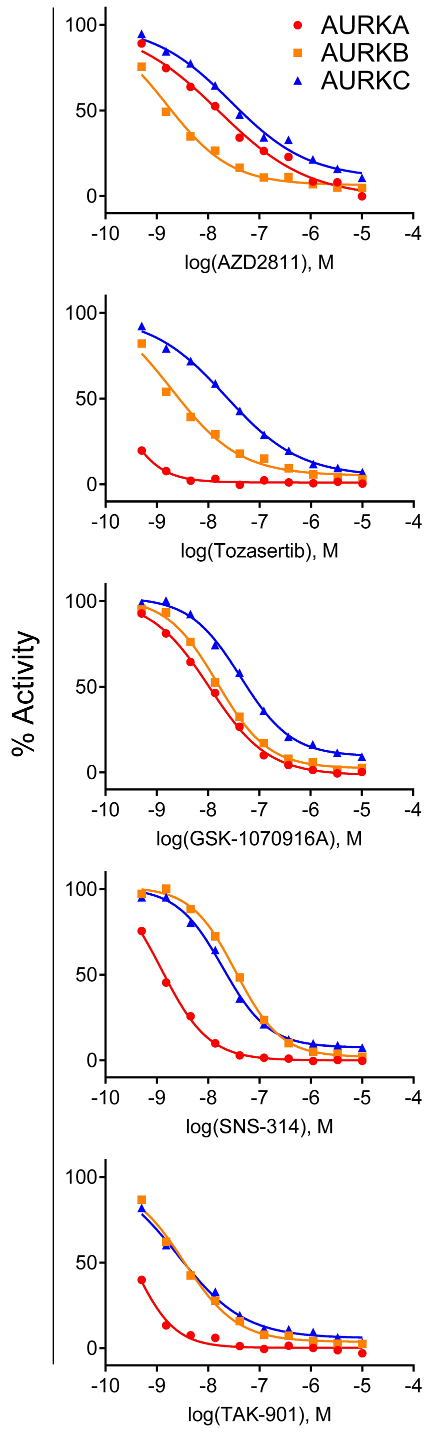

Supplementary Fig. S9. Biochemical profiling of aurora kinase inhibitors identified AZD2811 as a potent AURKB inhibitor. Activity of five aurora kinase inhibitors (AZD2811, tozasertib, GSK-1070916A, SNS-314, and TAK-901; n = 1) was tested against three AURK isoforms (AURKA, AURKB, and AURKC) and dose response curves were fit in GraphPad Prism using either three or four-parameter logistic equations. n = number of experimental replicates.

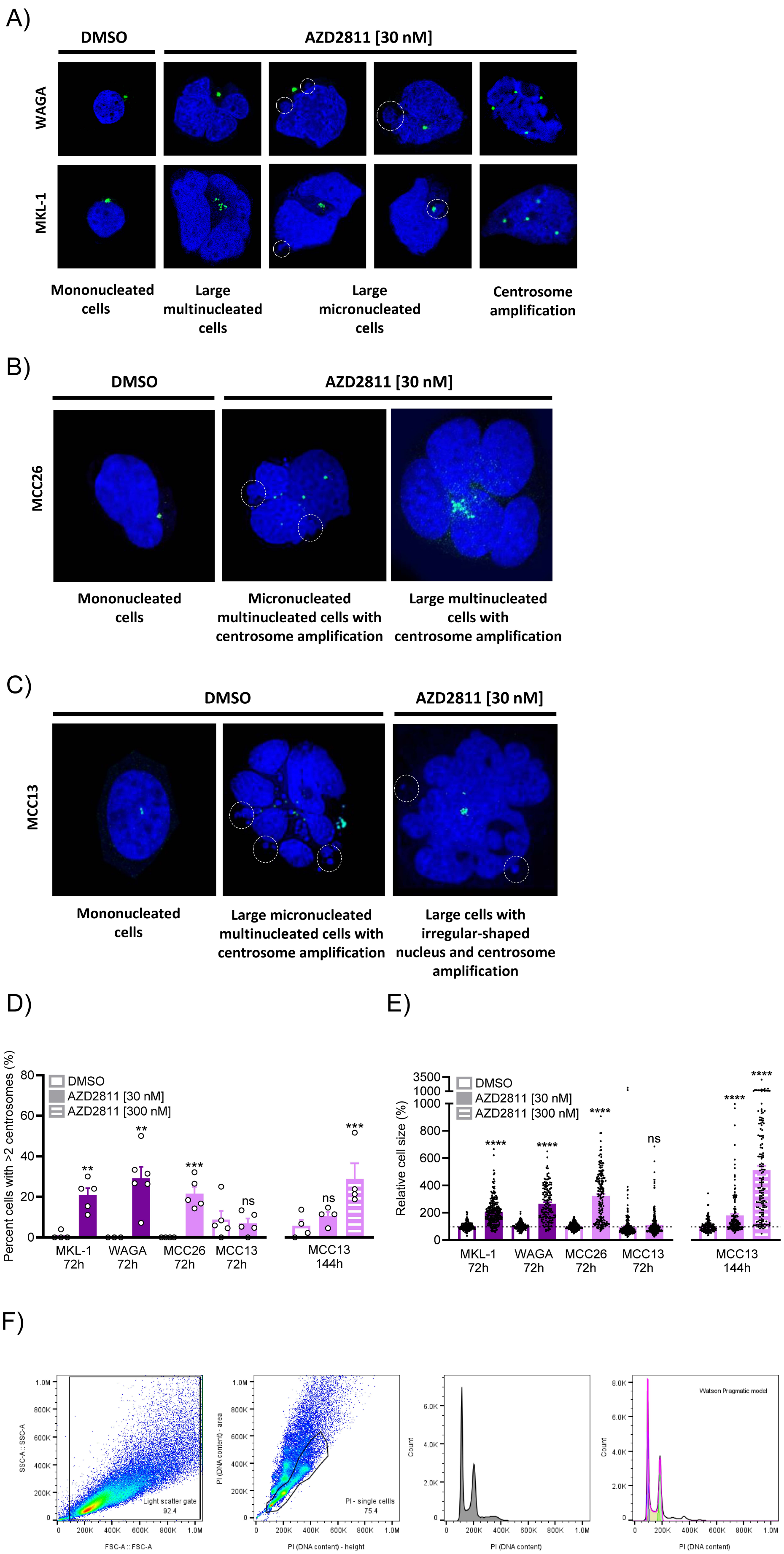
A)
DMSO
AZD2811 [30 nM]
WAGA
MKL-1
Mononucleated cells
Large multinucleated cells
Large micronucleated cells
Centrosome amplification
B)
DMSO
AZD2811 [30 nM]
MCC26
Mononucleated cells
Micronucleated multinucleated cells with centrosome amplification
Large multinucleated cells with centrosome amplification
C)
DMSO
AZD2811 [30 nM]
MCC13
Mononucleated cells
Large micronucleated multinucleated cells with centrosome amplification
Large cells with irregular-shaped nucleus and centrosome amplification
D)
Percent cells with >2 centrosomes (%)
DMSO
AZD2811 [30 nM]
AZD2811 [300 nM]
**
**
***
ns
ns
***
MKL-1 72h
WAGA 72h
MCC26 72h
MCC13 72h
MCC13 144h
E)
Relative cell size (%)
DMSO
AZD2811 [30 nM]
AZD2811 [300 nM]
****
****
****
ns
****
****
MKL-1 72h
WAGA 72h
MCC26 72h
MCC13 72h
MCC13 144h
F)
SSC-A :: SSC-A
FSC-A :: FSC-A
Light scatter gate
92.4
PI (DNA content) - area
PI (DNA content) - height
PI - single cellls
75.4
Count
PI (DNA content)
Watson Pragmatic model
Count
PI (DNA content)

Supplementary Fig. S10. AURKB inhibitor AZD2811 induces mitotic dysregulation in MCC. A) Immunofluorescent staining of MKL-1 and WAGA cells treated with AZD2811 30 nM for 72 h showing multinucleation, micronucleation, increased cell size, and centrosome amplification. B) Immunofluorescent staining of MCC26 cells treated with AZD2811 30 nM for 72 h showing multinucleation, micronucleation, increased cell size, and centrosome amplification. C) Immunofluorescent staining of MCC13 showing large multinucleated cells with centrosome amplification at baseline and large cells with irregular nuclei in response to treatment with AZD2811 30 nM for 72 h. D) Percent cells with > 2 centrosomes (n = 3; minimum 140 cells per treatment condition). E) Percent change in cell size (n = 3; minimum 200 cells per treatment condition). Unpaired two-sided t-test was used to determine statistical significance (*p < 0.05, **p < 0.01, ***p < 0.001, ****p < 0.0001). F) Gating strategy for flow cytometry cell cycle analysis shown in Figure 5C involved using a broad FSC (forward scatter) and SSC (side scatter) gate. This was followed by gating to exclude cell aggregates using a PI (propidium iodide) with H (height) versus A (area) or H (height) versus W (width) parameters. The PI area histogram was then analyzed in FlowJo using the Watson Pragmatic model. n = number of experimental replicates. For A, B, and C, DAPI (blue) labels the nuclei, pericentrin (green) labels the centrosomes, and dashed circles highlight micronuclei.

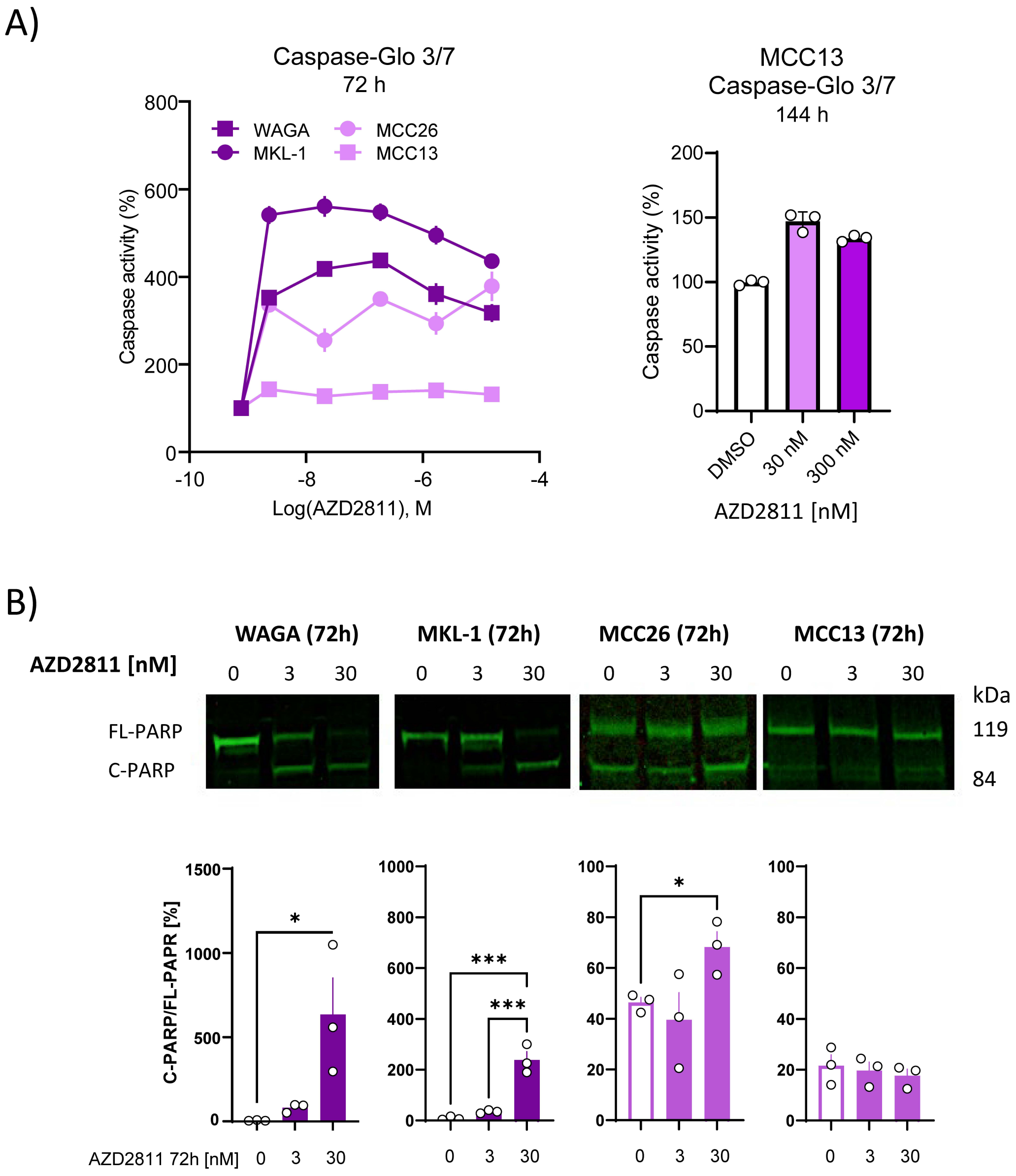

Supplementary Fig. S11. AURKB inhibitor AZD2811 induces apoptosis in MCC. A) Caspase 3/7 activity in AZD2811-treated MCC cell lines. B) Representative Western Blot demonstrating increased PARP cleavage in response to 72-h treatment with AZD2811 (n = 3) in WAGA and MKL-1 (VP-MCC) and MCC26 (VN-MCC) but not in MCC13 (VN-MCC). FL-PARP = full-length PARP and C-PARP = cleaved PARP. Unpaired two-sided t-test was used to determine statistical significance (*p < 0.05, **p < 0.01, ***p < 0.001). n = number of experimental replicates .

A)

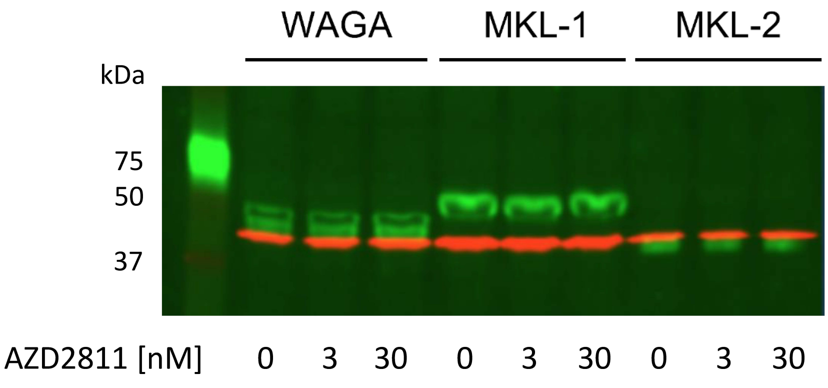

B)

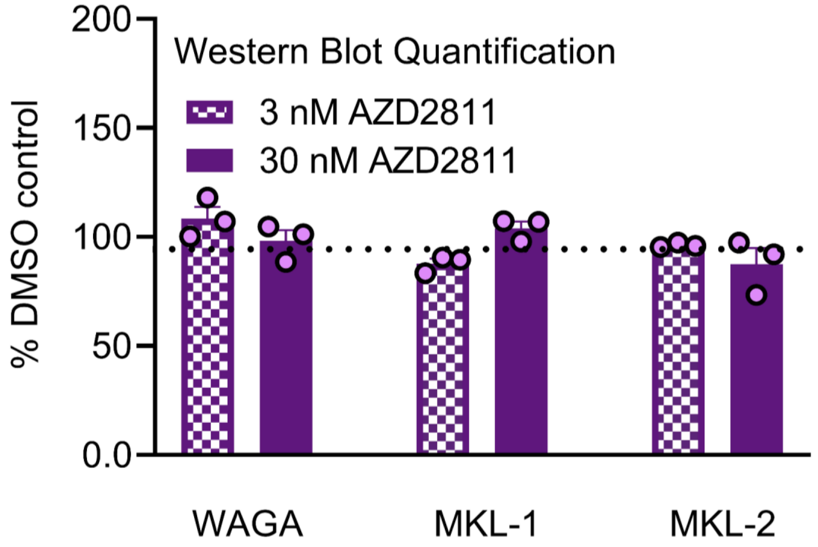

Supplementary Fig. S12. AURKB inhibitor AZD2811 has no effect on MCPyV-LT expression in VP-MCC cell lines. A) Representative Western Blot demonstrating no change in MCPyV-LT expression in 3 nM or 30 nM AZD2811-treated compared to DMSO-treated VP-MCC cell lines (WAGA, MKL-1, and MKL-2). B) Western Blot quantification (n = 3) demonstrating no change in MCPyV-LT expression (green) normalized to β-actin (red) in AZD2811-treated VP-MCC cell lines (WAGA, MKL-1, and MKL-2). n = number of experimental replicates.

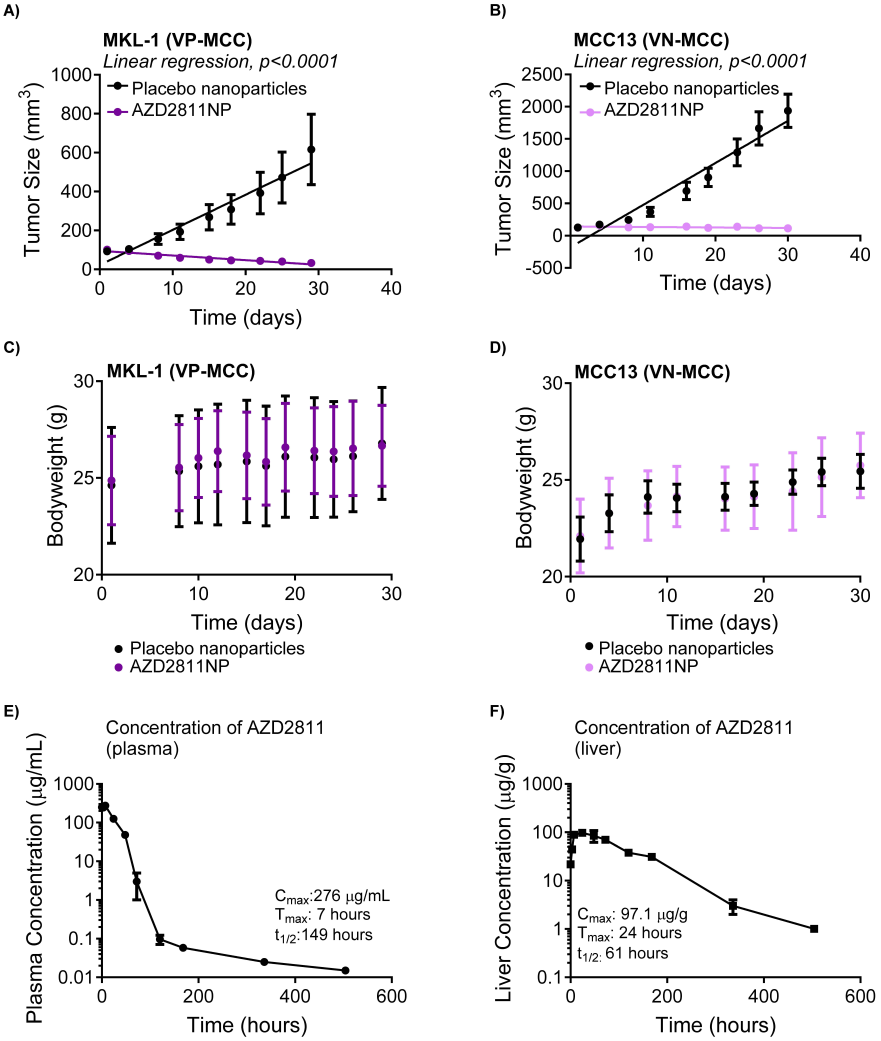

G)

	Plasma	Liver
$AUC_{0\rightarrow\infty}$(µg h/mL)	7980	13500
$t_{1/2}$ (h)	149	61
T_{max} (h)	7	24
C_{max} (µg/mL or µg/g)	276	97.1
Cl (mL/min/kg)	0.052	
Vss (L/kg)	0.060	

Supplementary Fig. S13. AZD2811NP tumor growth responses and pharmacokinetic profile. A/B) AZD2811NP significantly slowed MKL-1 (A) and MCC13 (B) tumor growth relative to placebo during treatment period as measured by linear regression ($p < 0.0001$; n = 10). C/D) Treatment with AZD2811NP did not reduce body weight in mice xenografted with MKL-1 (p = 0.71; n = 10) (C) or MCC13 (p = 0.9; n = 10) (D) cells relative to placebo nanoparticles as measured by linear regression in GraphPad Prism. Plasma (E) and liver (F) AZD2811 concentrations over time in female nude mice after a single 25 mg/kg IV dose of AZD2811NP (n = 3 animals for each time point). G) Pharmacokinetics summary after single IV administration of 25 mg/kg AZD2811NP. For A and B, n = number of tumors; for all other panels, n = number of animals.

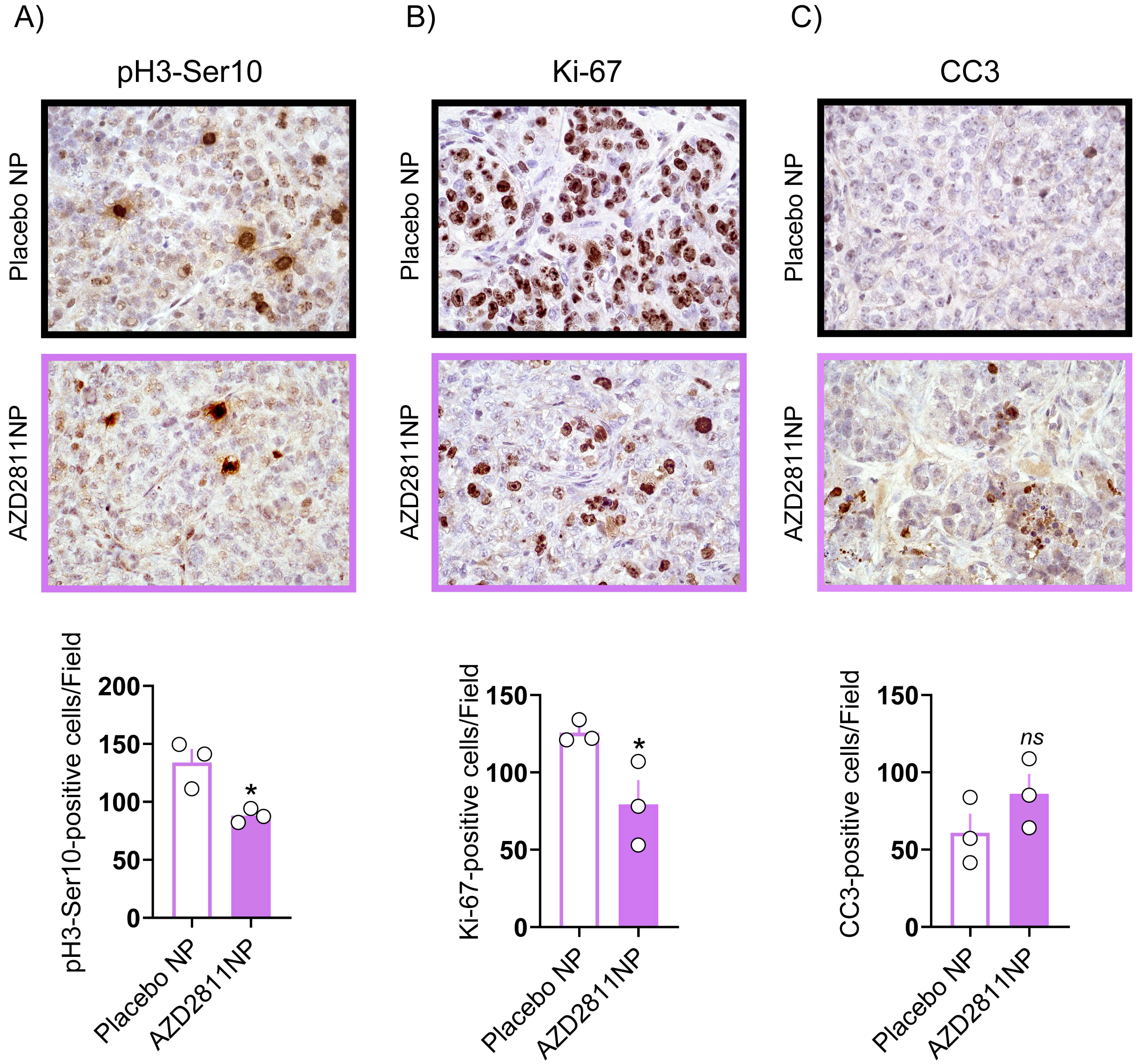

Supplementary Fig. S14. AURKB inhibitor AZD2811NP reduces H3Ser10 phosphorylation and Ki-67 expression in MCC13 xenografts. Immunohistochemical staining of AZD2811NP-treated MCC13 xenografts (n = 3) shows reduced H3Ser10 phosphorylation (A) and Ki-67 expression (B) with no significant change in caspase-3 cleavage (CC3) (C). Positive cells were blindly quantified from 4-6 20x images per tissue section. Unpaired two-sided t-test was used to determine statistical significance (*$p < 0.05$). n = number of animals.

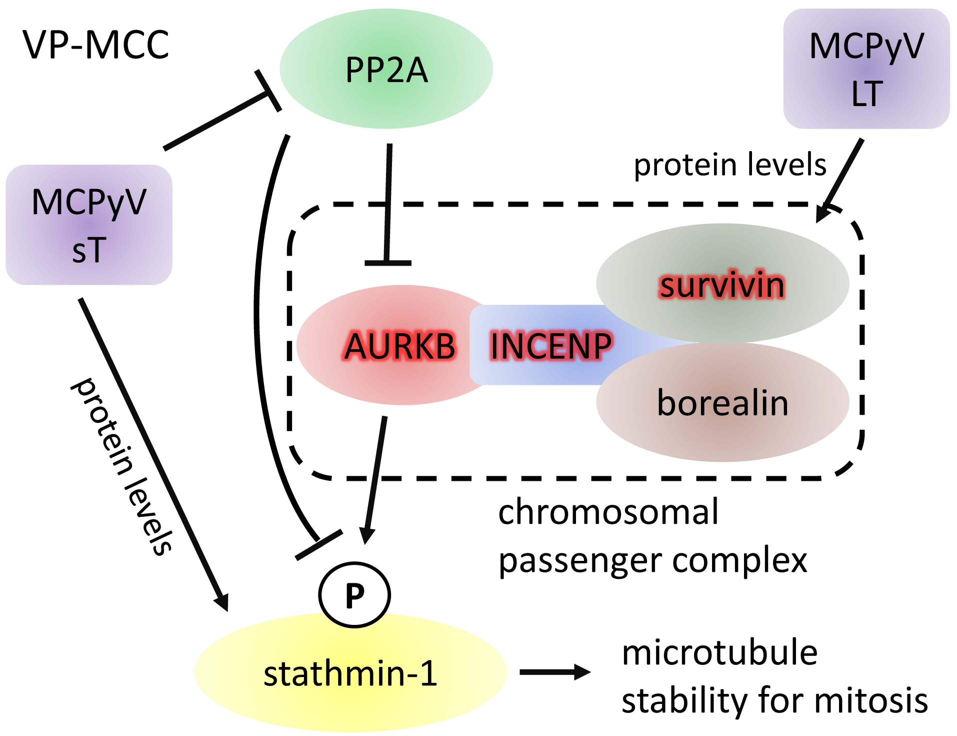

Supplementary Fig. S15. AURKB is a central hub connecting MCPyV oncogenes, the CPC, and stathmin-1. AURKB is part of the CPC along with INCENP, survivin, and borealin. The MCPyV-sT increases protein level of stathmin-1, which can be phosphorylated and regulated by AURKB. The MCPyV-LT increases expression of the CPC-member survivin. Together, the CPC and stathmin-1 regulate microtubule stability. Upregulation of CPC components by MCPyV oncogenes may make VP-MCC more dependent on CPC function. Consistent with this, inhibition of CPC members highlighted in red all cause selective reduction of VP-MCC viability.
