# Supplementary material for: High-throughput screening identifies Aurora kinase B as a critical therapeutic target for Merkel cell carcinoma
Source: Nat Commun. 2025 Feb 12;16:1583. doi: 10.1038/s41467-025-56504-7 (PMC11822212; doi:10.1038/s41467-025-56504-7)
Supplement: Supplementary file 2 — Description of Additional Supplementary Information [file 41467_2025_56504_MOESM2_ESM.docx]

**Description of Additional Supplementary Files**

File Name: Supplementary Data 1

Description: Compounds demonstrating activity in all MCC, VP-MCC, or VN-MCC cell lines, but not in control cell lines.

File Name: Supplementary Data 2

Description: List of pan-MCC, VP-MCC, and VN-MCC selective compounds relative to controls.

File Name: Supplementary Data 3

Description: Results of arrayed RNAi druggable genome screen in MKL-2 and MCC26 cells.

File Name: Supplementary Data 4

Description: MIPE library compound target subcategory annotation.

File Name: Supplementary Data 5

Description: Summary of high-throughput small molecule and RNAi screens.
