# Supplementary material for: High-throughput screening identifies Aurora kinase B as a critical therapeutic target for Merkel cell carcinoma
Source: Nat Commun. 2025 Feb 12;16:1583. doi: 10.1038/s41467-025-56504-7 (PMC11822212; doi:10.1038/s41467-025-56504-7)
Supplement: Supplementary file 8 — Reporting Summary [file 41467_2025_56504_MOESM8_ESM.pdf]

nature portfolio

Corresponding author(s): Isaac Brownell, MD, PhD

Last updated by author(s): Nov 27, 2024

Reporting Summary

Nature Portfolio wishes to improve the reproducibility of the work that we publish. This form provides structure for consistency and transparency in reporting. For further information on Nature Portfolio policies, see our Editorial Policies and the Editorial Policy Checklist.

Statistics

For all statistical analyses, confirm that the following items are present in the figure legend, table legend, main text, or Methods section.

n/a	Confirmed	
☐	☒	The exact sample size (*n*) for each experimental group/condition, given as a discrete number and unit of measurement
☐	☒	A statement on whether measurements were taken from distinct samples or whether the same sample was measured repeatedly
☐	☒	The statistical test(s) used AND whether they are one- or two-sided<br>*Only common tests should be described solely by name; describe more complex techniques in the Methods section.*
☒	☐	A description of all covariates tested
☐	☒	A description of any assumptions or corrections, such as tests of normality and adjustment for multiple comparisons
☐	☒	A full description of the statistical parameters including central tendency (e.g. means) or other basic estimates (e.g. regression coefficient) AND variation (e.g. standard deviation) or associated estimates of uncertainty (e.g. confidence intervals)
☐	☒	For null hypothesis testing, the test statistic (e.g. F, t, r) with confidence intervals, effect sizes, degrees of freedom and P value noted<br>*Give P values as exact values whenever suitable.*
☒	☐	For Bayesian analysis, information on the choice of priors and Markov chain Monte Carlo settings
☒	☐	For hierarchical and complex designs, identification of the appropriate level for tests and full reporting of outcomes
☒	☐	Estimates of effect sizes (e.g. Cohen's *d*, Pearson's *r*), indicating how they were calculated

Our web collection on statistics for biologists contains articles on many of the points above.

Software and code

Policy information about availability of computer code

Data collection	No software was used to collect data for this manuscript.
Data analysis	- qHTS was analyzed using software developed internally in NIH Chemical Genomics Center (http://tripod.nih.gov/curvefit/).<br>- GraphPad Prism was used to calculate IC50. All other drug activity analyses and comparisons were performed in R studio.<br>- Drug activity ranking in cancer cell lines was done in R studio (1.1.447)<br>- FLow cytometry analysis was done in FlowJo (10.10)<br>- WinNonLin software was used to calculate PK parameters (e.g. AUC, Cl, Vss, Cmax, Tmax, t1/2).<br>- All other experiments were analyzed by GraphPad Prism (8 or 10)

For manuscripts utilizing custom algorithms or software that are central to the research but not yet described in published literature, software must be made available to editors and reviewers. We strongly encourage code deposition in a community repository (e.g. GitHub). See the Nature Portfolio guidelines for submitting code & software for further information.

Data

Policy information about availability of data

All manuscripts must include a data availability statement. This statement should provide the following information, where applicable:

- Accession codes, unique identifiers, or web links for publicly available datasets
- A description of any restrictions on data availability
- For clinical datasets or third party data, please ensure that the statement adheres to our policy

Full data for the qHTS and arrayed RNAi screen are available at PubChem. qHTS: (https://pubchem.ncbi.nlm.nih.gov/, accession number 1296009) and RNAi screen: (https://pubchem.ncbi.nlm.nih.gov/, accession number pending). Other data will be shared upon reasonable request to the corresponding author.

Research involving human participants, their data, or biological material

Policy information about studies with human participants or human data. See also policy information about sex, gender (identity/presentation), and sexual orientation and race, ethnicity and racism.

Reporting on sex and gender	NA
Reporting on race, ethnicity, or other socially relevant groupings	NA
Population characteristics	NA
Recruitment	NA
Ethics oversight	NA

Note that full information on the approval of the study protocol must also be provided in the manuscript.

Field-specific reporting

Please select the one below that is the best fit for your research. If you are not sure, read the appropriate sections before making your selection.

☒ Life sciences ☐ Behavioural & social sciences ☐ Ecological, evolutionary & environmental sciences

For a reference copy of the document with all sections, see nature.com/documents/nr-reporting-summary-flat.pdf

Life sciences study design

All studies must disclose on these points even when the disclosure is negative.

Sample size	Sample size for high-throughput screen was n = 1. All other experiments included n ≥ 3.
Data exclusions	No primary data points were excluded except for when they failed the required criteria of the data analysis being applied as described in the methods section.
Replication	All experiments included biological replicates.
Randomization	Randomization was implemented whenever possible. For example, animals were randomized for the in vivo xenograft studies.
Blinding	Randomization was implemented whenever possible. For example, samples were blinded for immunofluorescence and immunohistochemistry quantification.

Reporting for specific materials, systems and methods

We require information from authors about some types of materials, experimental systems and methods used in many studies. Here, indicate whether each material, system or method listed is relevant to your study. If you are not sure if a list item applies to your research, read the appropriate section before selecting a response.

Materials & experimental systems

n/a	Involved in the study
	x Antibodies
	x Eukaryotic cell lines
x	Palaeontology and archaeology
	x Animals and other organisms
x	Clinical data
x	Dual use research of concern
x	Plants

Methods

n/a	Involved in the study
x	ChIP-seq
	x Flow cytometry
x	MRI-based neuroimaging

Antibodies

Antibodies used	- AURKB antibody (abcam: ab45145, lot# GR149373-2, 1:500)<br>- β-actin antibody (Santa Cruz Biotechnology: sc-47778, lot# I2116, 1:200)<br>- Stathmin-1 antibody (abcam: ab52630, lot# GR302254-6, 1:1000)<br>- phospho-Histone H3 (Ser10) antibody (MilliporeSigma: 06-570, lot# 2794867, 1:150)<br>- Histone H3 antibody (Cell Signaling Technology: 3638, lot# 8, 1:300)<br>- PARP antibody (Cell Signaling Technology: 9532, lot# 9, 1:1000)<br>- Pericentrin antibody (abcam: ab4448, lot# GR3425987-2, 1:2000)<br>- MCPyV large T antibody (Santa Cruz Biotechnology: sc-136172 (CM2B4), lot# DO524, 1:200)
Validation	- All antibodies are well published and validated by manufacturers for western blot , immunohistochemistry, or immunofluorescence.<br>- AURKB antibody (Henssen et al. 2017: doi: 10.18632/oncotarget.13440) and 19 other publications.<br>- β-actin antibody (Min et al. 2023: doi.org/10.1016/j.molcel.2023.06.016) and 14031 other publications.<br>- Stathmin-1 antibody (Morris et al. 2020: doi.org/10.1016/j.celrep.2020.02.079) and 26 other publications.<br>- phospho-Histone H3 (Ser10) antibody (ZuaZua-Villar et al. 2014: doi.org/10.1038/cddis.2014.231)<br>- Histone H3 antibody (Malone et al. 2024: doi.org/10.1126/sciadv.adm9449)<br>- PARP antibody (St. Louis et al. 2024: doi.org/10.1128/mbio.03429-23)<br>- Pericentrin antibody (Fu et al. 2015: doi.org/10.1083/jcb.201412109)<br>- MCPyV large T antibody (Gao et al. 2021: doi.org/10.1016/j.isci.2021.103264)

Eukaryotic cell lines

Policy information about cell lines and Sex and Gender in Research

Cell line source(s)	- WAGA, MKL-1, MKL-2, and UISO cells were obtained from Dr. Jürgen Becker<br>- MCC13 and MCC26 cells were obtained from Dr. Patrick Moore<br>- HaCaT, HEK293T, CRL-7250, and NIH-3T3 cells were obtained from Dr. Javed Khan
Authentication	All cell lines have been authenticated by IDEXX (CellCheckTM 16 Plus - Human)
Mycoplasma contamination	All cell lines tested negative for mycoplasma by IDEXX (CellCheckTM 16 Plus - Human)
Commonly misidentified lines<br>(See ICLAC register)	*Name any commonly misidentified cell lines used in the study and provide a rationale for their use.*

Animals and other research organisms

Policy information about studies involving animals; ARRIVE guidelines recommended for reporting animal research, and Sex and Gender in Research

Laboratory animals	Female athymic nude mice were obtained from Charles River (strain code: 553)
Wild animals	NA
Reporting on sex	In consideration of animal housing cost and available space, female athymic nude mice were used for in vivo experiments because they can be co-housed
Field-collected samples	NA
Ethics oversight	Animal studies were approved by the Animal Care and Use Committee of the National Cancer Institute – Frederick.

Note that full information on the approval of the study protocol must also be provided in the manuscript.

Plants

Seed stocks	NA
Novel plant genotypes	NA
Authentication	NA

Flow Cytometry

Plots

Confirm that:

- [x] The axis labels state the marker and fluorochrome used (e.g. CD4-FITC).
- [x] The axis scales are clearly visible. Include numbers along axes only for bottom left plot of group (a 'group' is an analysis of identical markers).
- [ ] All plots are contour plots with outliers or pseudocolor plots.
- [x] A numerical value for number of cells or percentage (with statistics) is provided.

Methodology

Sample preparation	Cells were washed in ice-cold PBS, and fixed in 75% ethanol for at least 24- hours. Following fixation, cells were stained with 50 ug/mL of propidium iodide (Biolegend) containing 100 ug/mL RNAse A (MilliporeSigm) per million cells for at least 3 hours at 4 °C before DNA content was measured.
Instrument	BD FACSCanto II (BC Biosciences) or Invitrogen Attune NxT Flow Cytometer (Life Technologies)
Software	FlowJo 10.10
Cell population abundance	No cell sorting was performed
Gating strategy	Supplementary Fig. S10F shows gating strategy for flow cytometry cell cycle analysis shown in Fig. 5C. It involved using a broad FSC and SSC gate. This was followed by gating to exclude cell aggregates using a PI (propidium iodide) with H (height) versus A (area) or H (height) versus W (width) parameters. The PI area histogram was then analyzed in FlowJo using the Watson Pragmatic model.

- [x] Tick this box to confirm that a figure exemplifying the gating strategy is provided in the Supplementary Information.
